# HES1, a target of Notch signaling, is elevated in canine osteosarcoma, but reduced in the most aggressive tumors

**DOI:** 10.1186/1746-6148-9-130

**Published:** 2013-07-01

**Authors:** Deanna D Dailey, Kristin P Anfinsen, Liza E Pfaff, EJ Ehrhart, J Brad Charles, Tina B Bønsdorff, Douglas H Thamm, Barbara E Powers, Thora J Jonasdottir, Dawn L Duval

**Affiliations:** 1 The Flint Animal Cancer Center, College of Veterinary Medicine and Biomedical Sciences, Colorado State University, Fort Collins, CO, USA; 2 Cell and Molecular Biology Graduate Program, Colorado State University, Fort Collins, CO, USA; 3 Department of Companion Animal Clinical Sciences, Norwegian School of Veterinary Science, P.O. Box 8416 Dep., Oslo, NO-0033, Norway; 4 Department of Microbiology, Immunology and Pathology, College of Veterinary Medicine and Biomedical Sciences, Colorado State University, Fort Collins, CO, USA; 5 University of Colorado Comprehensive Cancer Center, Aurora, CO, USA; 6 Department of Clinical Sciences, Animal Cancer Center, Colorado State University, 300 West Drake Road, Fort Collins, CO 80523-1620, USA

**Keywords:** Hes-1, HES1, Notch, Osteosarcoma, RT-PCR, RT-qPCR, Immunohistochemistry, Canine, Microarray

## Abstract

**Background:**

Hairy and enhancer of split 1 (HES1), a basic helix-loop-helix transcriptional repressor, is a downstream target of Notch signaling. Notch signaling and HES1 expression have been linked to growth and survival in a variety of human cancer types and have been associated with increased metastasis and invasiveness in human osteosarcoma cell lines. Osteosarcoma (OSA) is an aggressive cancer demonstrating both high metastatic rate and chemotherapeutic resistance. The current study examined expression of Notch signaling mediators in primary canine OSA tumors and canine and human osteosarcoma cell lines to assess their role in OSA development and progression.

**Results:**

Reverse transcriptase - quantitative PCR (RT-qPCR) was utilized to quantify *HES1, HEY1, NOTCH1 and NOTCH2* gene expression in matched tumor and normal metaphyseal bone samples taken from dogs treated for appendicular OSA at the Colorado State University Veterinary Teaching Hospital. Gene expression was also assessed in tumors from dogs with a disease free interval (DFI) of <100 days compared to those with a DFI > 300 days following treatment with surgical amputation followed by standard chemotherapy. Immunohistochemistry was performed to confirm expression of HES1. Data from RT-qPCR and immunohistochemical (IHC) experiments were analyzed using REST2009 software and survival analysis based on IHC expression employed the Kaplan-Meier method and log rank analysis. Unbiased clustered images were generated from gene array analysis data for Notch/HES1 associated genes.

Gene array analysis of Notch/HES1 associated genes suggested alterations in the Notch signaling pathway may contribute to the development of canine OSA. *HES1* mRNA expression was elevated in tumor samples relative to normal bone, but decreased in tumor samples from dogs with a DFI < 100 days relative to those with a DFI > 300 days. *NOTCH2 and HEY1* mRNA expression was also elevated in tumors relative to normal bone, but was not differentially expressed between the DFI tumor groups. Survival analysis confirmed an association between decreased HES1 immunosignal and shorter DFI.

**Conclusions:**

Our findings suggest that activation of Notch signaling occurs and may contribute to the development of canine OSA. However, association of low HES1 expression and shorter DFI suggests that mechanisms that do not alter HES1 expression may drive the most aggressive tumors.

## Background

Osteosarcoma (OSA) is the most common malignant bone tumor among children and adolescents with an incidence of 4.4 cases per million per year in the United States [1]. OSA is also the most common spontaneous primary bone tumor of dogs, estimated to affect greater than 8,000 dogs annually in the United States [2]. Tumor morphology, biological behavior, progression of disease and molecular characteristics are very similar in dogs and humans [2-7]. Consequently, dogs provide a valuable comparative model of human OSA. Standard of care therapy for both human and canine OSA patients remains a combination of surgery and chemotherapy, with five-year survival rates reported in humans as high as 70% [1,8] and median survival in canine patients around 200 days [2]. Unfortunately, in both human and canine patients approximately 80% are estimated to have micrometastases at presentation, some of whose tumors are also refractory to chemotherapy [2,8]. These patients continue to have a poor prognosis. Histologic classification alone has not proven clinically relevant for determination of tumors likely to metastasize or exhibit resistance to chemotherapy protocols. The focus of recent research, therefore, has turned toward molecular characterization of primary tumors, especially aberrant gene and/or protein expression that might correlate with prognosis or chemotherapy sensitivity.

Hairy and enhancer of split 1 (HES1), a basic helix-loop-helix (bHLH) transcriptional repressor, is a downstream target of the Notch signaling pathway. The intracellular domain of activated Notch receptors (NICD) translocates to the nucleus, forms a transcriptional activating complex with recombination signal binding protein for immunoglobulin kappa J region (RPBJκ) and activates expression of target genes including HES1 [9,10]. The HES1 protein contains both DNA-binding and protein-protein interaction domains important for its function as a transcriptional regulator (including negative regulation of its own transcription) [9,11,12]. Notch-independent HES1 expression can also result from Hedgehog and c-Jun N-terminal kinase (JNK) signaling as well as from RAS/MAPK signaling [10,13-15]. Regulation of HES1 expression and activity is dependent on the tissue, spatial and temporal factors, and the proteins with which it interacts [9,10].

Overexpression of Notch and/or HES1 is associated with a variety of human cancers including T-cell acute lymphoblastic leukemia (ALL), and ovarian, breast, cervical, prostate, colon and non-small cell lung cancers [16-19]. Notch/HES1 has also been shown to have tumor suppressor activity in some cancers including hepatocellular carcinoma, B-cell ALL, myeloid leukemia and neuroblastoma [20-23]. In human OSA, Notch is implicated in OSA cell proliferation, invasion and metastasis [24,25]. Increased HES1 mRNA expression was shown in some human OSA cells and OSA tumor samples compared to osteoblasts or normal bone and an association between high HES1 expression and decreased survival of OSA patients has been suggested [24-27]. Reduced invasiveness in response to suppression of Notch signaling and HES1 activity implicates Notch/HES1 signaling in metastasis [28]. Another study suggests both up-regulation of Notch and increased expression of HES1 in one OSA cell line occurs in response to activation of the Wnt/β-catenin pathway [29].

During bone development there is significant cross talk between the Wnt/β-catenin, hedgehog, and Notch pathways affecting osteoblast differentiation and maturation and influencing HES1 expression [10,29-31]. Like Notch and Wnt/β-catenin, aberrant hedgehog signaling is also associated with development of human cancers [31]. Previous studies in our lab identified decreased expression of three hedgehog pathway associated genes in OSA tumors from dogs with a disease free interval (DFI) < 100 days (poor-responders) compared with tumors from dogs with a DFI > 300 (good-responders) [32].

In order to explore the hypothesis that Notch signaling would be altered in canine OSA compared to normal bone samples, the current study examines the expression of NOTCH1 and 2 receptors and signaling targets, HES1 and HEY1, in canine OSA samples from patients with known outcome and normal bone tissues. Immunohistochemical analysis of HES1 protein was assessed in Kaplan-Meyer survival analysis to confirm the association of decreased HES1 expression with a shorter DFI.

## Methods

### Tumor donors

Chemotherapy-naïve primary tumor samples were selected from the Colorado State University (CSU) Flint Animal Cancer Center’s tissue archive. Samples are archived with owner consent and approval by the CSU Institutional Animal Care and Use Committee. Twenty tumors from good- and poor-responders (n = 10 each group) were selected following the protocol previously published [32]. Briefly, chemotherapy-naïve primary OSA samples were from dogs treated with surgical amputation followed by chemotherapy with doxorubicin and/or a platinum based drug (distribution of choice of drug was not significantly different between groups). All twenty dogs were free of thoracic metastases by radiographic analysis at diagnosis and follow up consisted of evaluation by clinical examinations including thoracic radiographs every 2–3 months after initial treatment. Disease free interval (DFI) was calculated from surgery until development of metastatic disease and samples were identified for cohorts of good responders (DFI > 300 days) and poor responders (DFI < 100 days) in order to flank the median DFI (200 days). Nine additional appendicular OSA tumor samples were collected from which matched normal metaphyseal bone was harvested from the same limb (at least one joint space away from the tumor) following amputation. These nine matched samples were collected at amputation as cases came in (convenience sample) and absence/presence of metastasis, post-operative treatment, and patient follow-up were less consistent in this population. Tumor and normal bone fragments collected at amputation were flash-frozen in liquid nitrogen and stored at -80°C. Tumor fragments were also fixed in 10% neutral buffered formalin for 24 hours with subsequent routine processing and paraffin embedding.

Immunohistochemical HES1 expression was also assessed in a subset of canine appendicular OSA patients from a previously reported multi-institutional randomized prospective clinical trial [33]. The study was approved by the Institutional Animal Care and Use Committees of the participating institutions. All dogs underwent amputation followed by 5 cycles of adjuvant doxorubicin, with or without an investigational matrix metalloprotease inhibitor. Inclusion/exclusion criteria, staging, and follow-up procedures were standardized and tumor tissues were processed as previously reported [33]. Histologic grading (from 1 to 3) was performed by one author (BEP) utilizing a schema incorporating amount of matrix, percent necrosis, nuclear pleomorphism, nucleolar size/number and mitosis score [33]. Mitotic index was calculated by counting the number of mitotic figures per 10 random 400× fields.

### Cell culture

Canine cell lines used in this study were provided by Dr. Douglas Thamm; all cell lines were validated for species and genetic identity using short-tandem-repeat (STR) profiling as previously described [34]. Human OSA cell lines were obtained from Dr. Douglas Thamm (MG63, SAOS-2, SJSA-1), Dr. Hue Luu (MG63.2), or purchased from ATCC (U2OS). The MG63.2 cell line is a metastatic sub-line of the MG63 line, obtained via serial passage of rare lung metastases from MG63 [35]. All non-purchased cell lines were validated prior to use using STR profiling by the University of Colorado DNA Sequencing Shared Resource. Cells were cultured in C10 media (DMEM high glucose with 4 mM L-glutamine (Hyclone Laboratories, Inc.), 1 mM of sodium pyruvate, 2× MEM vitamins, 1× MEM non-essential amino acids, 1× antibiotic-antimycotic (100×: 10,000 IU/ml penicillin, 10,000 ug/ul streptomycin and 25ug/ml) (all additives from Mediatech, Inc.), and 10% fetal bovine serum (FBS) (Atlas Biologicals, Fort Collins, CO).

### RNA extraction

Total RNA was extracted from tumors and RT-qPCR was conducted as described previously [32]. Briefly, samples were freeze-fractured, homogenized, extracted with Trizol reagent (Invitrogen, Carlsbad, CA) and purified with RNeasy clean up (Quiagen, Valencia, CA) following manufacturer’s protocols. RNA was extracted from normal bone using the same protocol with an additional spin of 800× *g* at 4°C for 5 minutes following homogenization. The supernatant was carried forward through the Trizol protocol. Total RNA was extracted from human and canine OSA cells using the RNeasy Kit (Qiagen) per the manufacturer’s protocol. RNA was quantified via spectrophotometry and bioanalyzed for integrity as described in O’Donaghue et al. [32] with samples used having a RNA integrity number of at least 8. Human adult osteoblast total RNA was purchased from CELL Applications, Inc.

### Reverse transcriptase PCR and quantitative real time PCR

cDNA synthesis was completed using the QuantiTect Reverse Transcription Kit (Qiagen) with 1 or 3 μg input RNA. RT-qPCR of cDNA was run using iQ SYBR Green Supermix (Bio-Rad) and 25 ng equivalent RNA input in 25 μL reactions on a Stratagene Mx3000P instrument. Expression in canine cells and tissues was normalized to hypoxanthine phosphoribosyltransferase 1 (*HPRT1*) expression. *HPRT1* was selected based on its consistent moderate expression in our sample sets in prior microarray and RT-qPCR analysis (see Additional file 1 and reference [32]) and its previous use as a canine reference gene [36]. Consistent with current recommendations for the selection of reference genes and because no single reference gene exhibited unchanged expression between samples, expression in human OSA cells was normalized to the geometric mean of four reference genes; ribosomal protein S15 (*RBS15*), glyceraldehyde-3-dehydrogenase (*GAPDH*), 18S ribosomal RNA (*18SrRNA*) and *HPRT1*[37]. Primer sequences and efficiencies for all genes and the full sequence of the canine *HES1* amplicon are listed in Additional file 2. Primers were designed using Primer-Blast based upon NCBI RefSeq mRNA sequences when available. Primers were designed to be intron spanning when possible and cross-checked for specificity via UCSC in silico PCR. Primers were further validated with standard curves to calculate efficiency, and dissociation curves as previously described [34]. RT-qPCR products were validated for size by agarose gel electrophoresis and sequenced to confirm identity. The 161 bp canine *HES1* amplicon revealed 98% homology to the human homolog of *HES1*. Human *HES1* primers used were the same as those used by Zhang et al. [24]. The identity of the 200 bp amplicon was verified as human *HES1* by dideoxy sequencing (CSU DNA sequencing Core).

### Western blot

Western blot analysis was performed on canine and human OSA cells using whole cell lysates or cytoplasmic and nuclear fractions. Whole cell lysates were prepared in triethanolamine (TEA) lysis buffer (55 mM TEA, pH 7.5, 111 mM NaCl, and 2.2 mM EDTA, 0.44% SDS) with 1× Complete Protease Inhibitor Cocktail (Roche Diagnostics). Protein concentrations were determined using the bicinchoninic acid (BCA) protein assay (Thermo Scientific). Nuclear extracts were prepared using a hypotonic 0.5% or 0.25% IgePal (NP-40) buffer (10 mM Hepes, 1.5 mM MgCl, and 10 mM KCl). Briefly, harvested cell pellets were re-suspended in IgePal buffer with protease inhibitor while vortexing, incubated on ice for 0–5 minutes, and centrifuged for 5 minutes at 500× *g*. The supernatant (cytoplasmic fraction) was collected and the pellet (nuclear fraction) was re-suspended in TEA lysis buffer with protease inhibitors. Samples were separated using SDS-PAGE and transferred to a polyvinylidine fluoride membrane. The membrane was blocked with 5% non-fat dry milk (NFDM) for one hour at room temperature and incubated with rabbit monoclonal anti-HES1 antibody (RabMAb EPR4226, 1:500; Epitomics) in 5% bovine serum albumin (BSA) at 4°C overnight. After washing in 0.1% Tween 20-Tris-buffered saline (TBST) the membrane was incubated with secondary horseradish peroxidase conjugated goat anti-rabbit antibody (1:5000; Bio-Rad) in 5% NFDM for one hour at room temperature. SuperSignal West Dura Extended Duration Substrate (Pierce Biotechnology) was used to detect chemiluminescent signals. Band intensity from four experiments using whole cell lysates from MG63 and MG63.2 cell lines were analyzed using ImageJ software. The intensity of the HES1 band was normalized to the corresponding α-tubulin loading control.

### Immunohistochemistry (IHC)

IHC to detect HES1 expression was performed on 4 μm sections from formalin-fixed paraffin embedded (FFPE) tumor tissues using standard immunoperoxidase techniques on charged slides with hematoxylin counter stain. Slides with sections were heated at 60°C for 30 minutes, allowed to cool, and deparaffinized with xylene or a citrus based clearing solution (Thermo-Fisher Scientific), and rehydrated with descending ethanol concentrations in deionized water (100%, 95%, 75% and 50%). Heat induced epitope retrieval was done with 10 mM sodium citrate buffer (pH 6.0) heated in a pressure cooker for 1 minute at 125°C. Endogenous peroxidase activity was blocked with 3% hydrogen peroxide at room temperature for 5 minutes with 3 washes in TBST both before and after. Slides were incubated with a non-serum protein block (Background Sniper, Biocare Medical) at room temperature for 15 minutes followed by incubation with primary antibody overnight at 4°C overnight. The primary antibody (anti-HES1 RabMAb, Epitomics) was used at a dilution of 1:750 (diluted in Antibody Diluent, Dako). Sections were then incubated with a prediluted secondary antibody conjugated to horseradish peroxidase (Envision and Dual Link System HRP, Dako) for 30 minutes at room temperature with 3 TBST washes both before and after. Diaminobenzidine (DAB, Ventana Medical Systems) was used as a chromogen for immunoreactive complex detection and slides were counterstained with hematoxylin.

Sixty-one additional FFPE tumor samples were analyzed for HES1 immunohistochemical expression utilizing a protocol similar to that described above with the following exceptions: primary antibody was diluted in 2.5% normal goat serum in TBST (1:750 or 1:375, higher antibody concentration was used in subsequent batches to increase immunoreactivity signal), and detection was performed using biotinylated anti-rabbit IgG antibody in a Vectastain ABC Kit (Vector Laboratories). The IHC was performed in five batches of 8 to 18 slides each with the same antibody dilution used for an entire batch. Variations in antibody dilutions were controlled for by inclusion of a positive control tumor slide with a total immunoreactivity score of 4 (percent cells staining score of 2 and intensity score of 2; Table 1). All samples within each batch were scored in reference to the control. Negative controls lacking primary antibody were included in each batch.

**Table 1:** Summary of data for dogs with DFI > 300 and DFI < 100 days, including HES1 immunohistochemistry score

**Breed**	**Age at Dx (yrs)**	**Sex**	**Tumor Loc**	**DFI (days)**	**Avg% stain**	**Avg stain intensity**	**Total score**
Greyhound	4.4	MC	PH	40	1	1	1
Rottweiler	5	MC	DF	69	3	3	9
Greyhound	7	MC	DF	77	2	1	2
Mix	9	FS	T	90	2	1	2
Greyhound	8	FS	PT	94	1	2	2
Labrador	10.2	FS	DH	95	3	3	9
Mix	8.8	MC	DF	97	2	1	2
Golden	10.8	MC	PH	97	2	1	2
Mix	7.6	FS	DR	307	2	2	4
Greyhound	7.1	MC	PH	467	1	1	1
Mix	12.4	MC	DR	694	3	3	9
Malamute	10.1	FS	DR	734	3	2	6
Labrador	8.7	MC	T	787	3	3	9
Golden	8	FS	DR	885	3	2	6

*DFI* disease free interval, *Dx* diagnosis, *MC* male castrated, *FS* female spayed, *P* proximal, *D* distal, *H* humerus, *R* radius, *T* tibia, Total Score is product of scores for % cells staining and staining intensity.

HES1 antibody validation was done using human placenta and canine lung and pancreas as positive control tissues. Specificity of the primary antibody was verified using a HES1 blocking peptide (Epitomics). Briefly, primary antibody was incubated with 25× (by mass) blocking peptide in antibody diluents (at both 1:375 and 1:750) for one hour at room temperature before application to canine control and sample tumor slides. Positive and negative controls with sections from the same tissues were incubated in parallel.

Immunohistochemical scoring of all slides was performed independently by two authors blinded to case information. A positive cell was any neoplastic cell with distinct brown staining in the nucleus (stromal cells and endothelial cells were not counted). The percentage of positive cells in each sample was estimated based on an average of two or more high powered fields and scored as follows, 1: < 50% cells stain positive, 2: 50-75% cells stain positive, 3: > 75% cells stain positive. Average stain intensity ranged from 1 to 3 (lowest to highest intensity). Field location and number were selected randomly at the discretion of the individual scorer. The product of the percentage and intensity scores made up the overall immunoreactivity score (ranging from 1 to 9). Both scorers simultaneously reviewed slides with conflicting scores (scores deviating by more than 1 in either category) (n = 5) and consensus was reached. After review, total scores were averaged for statistical analyses.

### Immunocytochemistry (ICC)

Immunocytochemistry was performed utilizing the same reagents and a similar protocol to that used for IHC. Slides were prepared via cytospin and dried overnight. Prior to the blocking step cells were fixed with 100% methanol at room temperature for 15 minutes, allowed to dry, washed in TTBS and incubated in 0.1% TritonX-100 in TBS for 7–12 minutes. The remainder of the procedure was identical to that used for IHC, but a higher concentration of primary antibody (1:250) was used.

Photomicrographs (IHC and ICC) were taken using the Olympus BX51 Research System Microscope with an Olympus dp70 Digital Camera System. Minimal additional editing was done in Microsoft ^®^ PowerPoint ^®^ for Mac 2011.

### Gene expression microarray analysis

Total RNA from primary OSA tumor samples from dogs with DFI < 100 (n = 8) and DFI > 300 (n = 7) was analyzed on GeneChip Canine 2.0 Genome Arrays (Affymetrix, Santa Clara, CA) at CSU’s Rocky Mountain Regional Center for Excellence (RMRCE) Genomics Core per Affymetrix protocols as described [35]. Normal bone samples (n = 8) were analyzed using an identical protocol. Samples used for microarray analysis were a subset of those used for RT-qPCR (microarray samples were limited due to array costs). Microarray pre-processing combining the osteosarcoma samples with the normal bone samples was conducted using Probe Logarithmic Intensity Error (PLIER) estimation algorithms with log_2_ transformations. Probesets including Notch receptor ligands, effectors, or targets of either the canonical Notch pathway or HES1 were selected based on literature review, Ingenuity**^®^** Systems Pathway analysis, and/or inclusion in The Human Notch Signaling Pathway RT^2^ Profiler™ PCR Array (SAbiosciences) (Additional file 1). CIMminer was used to generate clustered images of the data from the 75 selected probesets with unsupervised clustering on both axes and the following parameters: average linkage, Euclidean distance, and quantile binning with median centering of the data. Full microarray data for the DFI groups is available through NCBI’s Gene Expression Omnibus (GEO) via accession number GSE24251.

### Statistics

Statistical analysis of RT-qPCR and immunohistochemistry data (not including survival data) was performed using Prism software (GraphPad Software, La Jolla, CA). For RT-qPCR data standard curves, dissociation curves and amplification data was collected on a Stratagene Mx3000P instrument and analyzed using the Rest2009 software [38]. *HES1* RT-qPCR data was also analyzed using the 2^(-ΔΔCt)^ method [39] with similar results. IHC scores for the DFI > 300 and DFI < 100 tumors were analyzed with a 2-tailed Fischer’s exact test after separating scores into low expression (total score less than 4) and high expression (total score greater than or equal to 4) categories. The cut off was based on results of receiver-operating characteristic (ROC) analysis of immunohistochemical scores for the DFI > 300 and DFI < 100 groups. Welch *t*-test in ArrayTrack 3.5.0 with false discovery rate correction for multiple comparisons (FDR; based on all array probesets) was used to compare microarray gene expression data. Significance was defined as p < 0.05 (Welch *t*-test) or q < 0.05 (FDR).

Statistical analysis of survival data was performed using a combination of Prism and SPSS software version 20 for Macintosh (IBM, Armonk, NY). Correlations between HES1 expression levels and other markers on a continuous scale were evaluated using linear regression analysis. A 2-tailed, unpaired *t*-test was used to evaluate the association between HES1 expression levels and categorical markers. The median DFI was estimated using the Kaplan-Meier method, and comparisons between groups made using log rank analysis for categorical variables. For continuous variables, markers were categorized into a low and high group using the median value as the break point. Multivariable Cox regression analysis was then performed, utilizing both forward and backward stepwise models. Variables identified with a univariate p-value of <0.1 were included in the multivariate analysis. For all other tests, p-values of <0.05 were considered significant.

## Results

### Gene expression analysis of Notch/HES1-associated genes groups normal and OSA bone samples, but does not distinguish DFI groups

**Figure 1:**
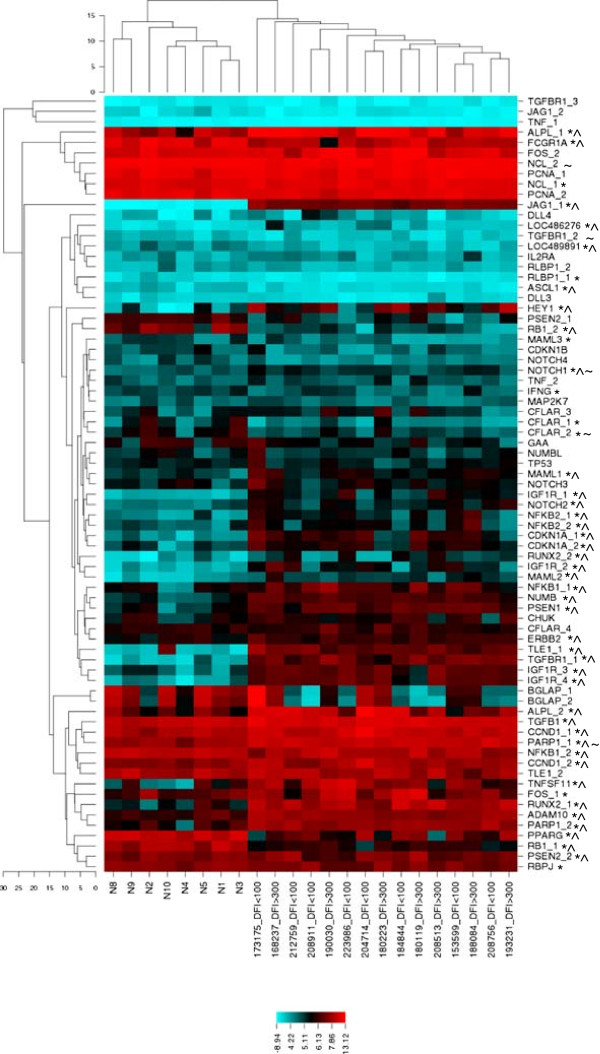
**Differential expression of Notch/HES1-associated genes in canine osteosarcoma.** Unbiased cluster analysis separates normal bone from tumors, but does not discriminate DFI < 100 day and DFI > 300 day primary tumors groups. An asterisk (*) and a caret (^) denote genes significantly different between tumor and normal bone (* p < 0.05, ^ q < 0.05). Genes different between DFI groups (p < 0.05) are denoted by (~). Multiple probesets are present for some genes. LOC486276 = Deltex 1 homolog (DTX1), LOC489891 = LFNG O-fucosylpeptide 3-beta-N-acetylglucosaminyltransferase/lunatic fringe (LFNG). Colored bar below indicates the intensity scale of log^2^ transformed expression values.

To assess the biological relevance of Notch/HES1 signaling in canine osteosarcoma, probesets including Notch receptor ligands, effectors, or targets of either the canonical Notch pathway or HES1 were selected from Canine 2.0 gene array data and analyzed for differential gene expression as described in materials and methods. Unbiased cluster analysis of data for the 51 Notch/HES1-associated genes separated normal bone from tumors, but did not discriminate between the DFI groups (Figure 1). In total, 30 of 51 (58.8%) Notch/HES1 pathway associated genes examined were significantly different between tumor and normal bone (p < 0.05, q < 0.05); 23/30 (76.7%) had increased expression in tumors. Specifically, mRNA expression of *NOTCH1* and *NOTCH2* was elevated in tumor samples compared to normal bone (p < 0.05, q < 0.05). None of the genes evaluated had significantly different expression between DFI groups when corrected for multiple comparisons. *HES1* was not included on the Canine 2.0 chip, but *HEY1*, another Notch target, was also elevated in tumors compared to normal bone (p < 0.05, q < 0.05).

**Figure 2:**
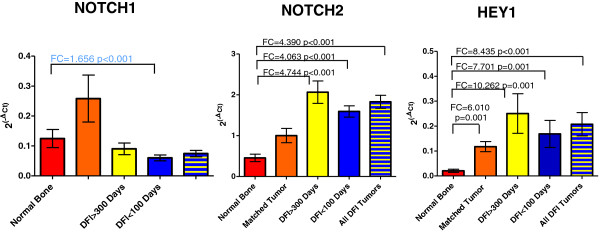
**Expression of *NOTCH1*, *NOTCH2 *and *HEY1 *mRNA in canine normal bone and osteosarcoma (RT-qPCR).***NOTCH1*, *NOTCH2,* and *HEY1* mRNA expressed as 2^(-ΔCT)^ normalized to *HPRT1* is shown for normal bone (n = 9), matched tumors (n = 9), tumors from dogs with DFI > 300 days, tumors from dogs with DFI < 100 days, and combined DFI group tumors. Comparisons of each tumor group relative to normal bone and DFI < 100 relative to DFI > 300 day groups were analyzed with REST 2009 software and significant fold changes are indicated by brackets on the graph. Values in blue indicate the reduced fold-change expression in DFI < 100 compared to normal bone. Bars represent mean ± SEM.

**Figure 3:**
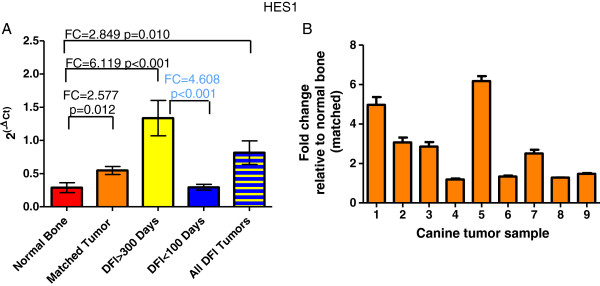
**Expression of *HES1 *mRNA in canine normal bone and osteosarcoma (RT-qPCR).** (**A**) *HES1* mRNA expressed as 2^(-ΔCT)^ normalized to *HPRT1* in normal bone (n = 9), matched tumors (n = 9), tumors from dogs with DFI > 300 days (n = 10), tumors from dogs with DFI < 100 days (n = 10), and combined DFI group tumors. Comparisons of each tumor group relative to normal bone and DFI < 100 relative to DFI > 300 day groups were analyzed with REST 2009 software and significant fold changes are indicated by brackets on the graph. Values in blue indicate reduced fold-change in DFI < 100 relative to DFI > 300 group. (**B**) Fold change in expression calculated using the comparative Ct (2^(-ΔΔCt)^) method between each canine tumor and its matched normal bone sample (normalized to *HPRT1*). Bars represent mean ± SEM.

RT-qPCR analysis for *NOTCH1, NOTCH2, HEY1 and HES1* was conducted on the normal bone/matched OSA and DFI tumor sample sets (Figures 2 and 3). *NOTCH1* exhibited decreased expression in the DFI < 100 day group relative to normal bone (FC down – 1.656, p < 0.001), with no other significant changes measured. This result differed from the 1.27 fold upregulation of *NOTCH1* identified in the gene array analysis*,* however previous studies have shown that fold-change differences <1.5 are frequently unreliable [40]. Consistent with the array data, *NOTCH2* exhibited an approximate 4-fold elevation in expression in both sets of DFI tumors, separately and in combination, relative to normal bone (p < 0.001). Similarly, *HEY1* expression was elevated in each tumor group by a fold-change ranging from 6 to 10.2 (p ≤ 0.001). RT-qPCR analysis of these Notch signaling pathway elements confirmed our finding that Notch signaling is elevated in tumors relative to normal bone, but not between tumors in the two DFI groups.

### HES1 mRNA expression in tumors and its prognostic significance

RT-qPCR was also used to assess *HES1* mRNA levels in OSA tumor and matched normal bone samples. Average *HES1* mRNA expression was elevated 2.57-fold in canine OSA tumors compared to the matched normal bone (Figure 3A; p = 0.012); however, this fold change was highly variable when each OSA tumor was compared to its matched normal bone sample, with 5 tumors exhibiting elevated expression compared to normal bone and 4 tumors having virtually unchanged expression (Figure 3B, range 1.19-6.17-fold).

We also assessed mRNA levels for *HES1* in tumors taken from dogs with a DFI <100 days or DFI >300 days following treatment by amputation and chemotherapy. We found that *HES1* expression was elevated 4.608-fold in the DFI > 300 tumors compared to the DFI < 100 group (Figure 3A; p < 0.001). *HES1* expression in the DFI < 100 group was not different from the normal bone samples.

**Figure 4:**
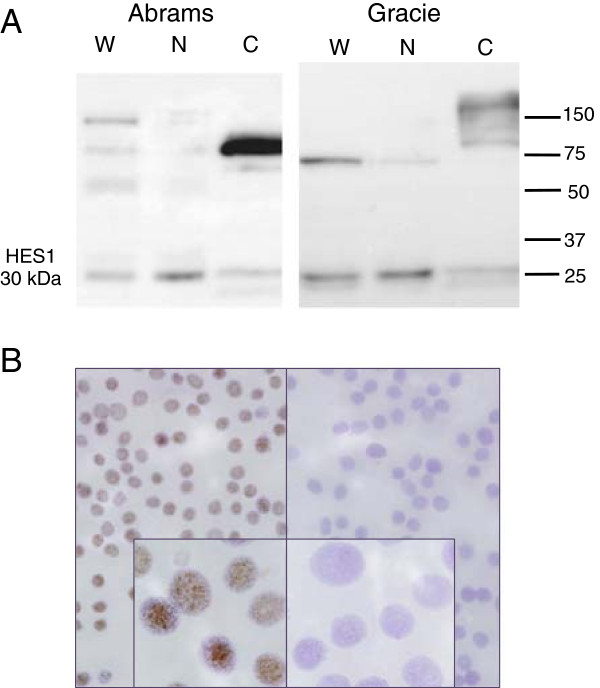
**Western blot and immunocytochemistry (ICC) results assessing HES1 expression in canine osteosarcoma cells.** (**A**) Western blot analysis of whole cell (W), nuclear (N) and cytoplasmic (C) fractions of canine osteosarcoma Abrams and Gracie cell lines. A 30 kDa band (HES1) is present in whole cell and enriched in extracted nuclear lysates. Larger non-specific bands are enriched in the cytoplasmic fractions. Equal amounts of total protein were loaded in each lane. (**B**) ICC shows nuclear staining for HES1 in canine OSA cells (Gracie). Panel on the right is the secondary-only negative control. Photomicrographs were taken at 20× and 100× (oil, inset) magnification; haemotoxylin counterstain.

**Figure 5:**
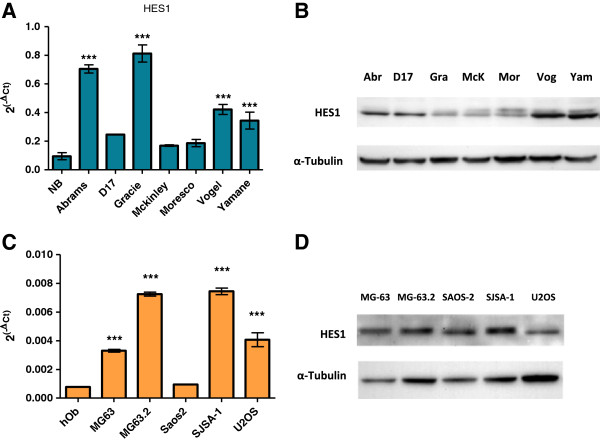
**Expression of *HES1 *mRNA and protein in human osteosarcoma cell lines.***HES1* mRNA in canine OSA cell lines and normal canine bone tissue (NB) expressed as 2^(-ΔCT)^ normalized to *HPRT1* (**A**). *HES1* mRNA in human OSA cell lines and normal human osteoblasts expressed as 2^(-ΔCT)^ normalized to the geometric mean of *RBS15, GAPDH, 18SrRNA,* and *HPRT1* (**C**). Data are graphed as mean ± SEM, *** P < 0.001, ** P < 0.01, Two-way ANOVA with Dunnett’s Multiple Comparison Test. (**B** and **D**) Western blot shows characteristic distinct HES1 band at 30 kDa. Blot was stripped and re-probed with an antibody against α-tubulin to serve as a protein loading control.

Messenger RNA levels of HES1 were measured in canine and human osteosarcoma cell lines and confirmed using Western blot analysis using a rabbit monoclonal anti-human HES1 antibody as described to determine if *HES1* mRNA levels correlated to protein expression, (Figures 4 and 5, Additional file 3). Comparison of canine and human amino acid sequence of the *HES1* gene identified 86% homology in the epitope targeted by this antibody. This was based on the predicted amino acid sequence of NCBI reference sequence XM_548669.1, which has been removed as a result of standard genome annotation processing. No additional canine *HES1* record is currently available. Western blot analysis of whole cell OSA cell lysates revealed a 30 kD protein (HES1) as well as larger non-specific bands (Figure 4A, W). Given the role of HES1 as a transcriptional regulator, we hypothesized that active HES1 protein would reside in the nucleus. Western blot analysis of isolated nuclear and cytoplasmic fractions from both canine and human OSA cell lines confirmed enrichment of the 30 kD HES-1 protein in the nuclear fraction (Figure 4A, N) while the non-specific bands were enriched in the cytoplasm fraction (Figure 4A, C). Since equal amounts of total protein were loaded in each lane, the increased intensity and/or number of nonspecific bands in the cytoplasmic fraction were likely the result of concentration of these cytoplasmic proteins relative to total protein. Experiments using human OSA cells showed similar results (Additional file 3).

*HES1* mRNA and protein expression varied between cell lines in both canine and human OSA cells (Figure 5). For human cell lines mRNA expression was similar to that previously published [24,25]. In general, *HES1* mRNA expression was increased in canine cell lines relative to normal canine bone tissue (Figure 5A) and in human OSA cell lines relative to human osteoblasts (Figure 5C). Western blot analysis showed a characteristic band at 30 kDa with variable expression between cell lines (Figure 5B and 5D). Interestingly, the metastatic subline of MG63 cells, MG63.2, exhibited elevated levels of mRNA compared to the MG63 line, but protein expression was not significantly different between the two lines (Additional file 4).

We validated immunoreactivity using FFPE human placenta and found positive strong nuclear and cytoplasmic staining of placental macrophages (Hafbauer cells), moderate nuclear +/- cytoplasmic staining of stromal cells and light nuclear staining of endothelial cells consistent with Notch activity in placenta reported by Herr et al. [41]. Staining of additional canine control tissues revealed positive punctate to diffuse intranuclear staining of pancreatic cells, endothelial cells and subsets of pulmonary epithelial cells as described in human literature [42-44] (see Additional file 5). Addition of a blocking peptide specific for the epitope targeted by our antibody eliminated all staining (data not shown). Immunocytochemistry of canine OSA cells (Gracie) showed diffuse nuclear staining consistent with the specific 30 kDa protein identified in the nuclear lysate by western analysis (Figure 4B).

### Increased immunohistochemical HES1 staining is associated with increased disease free interval

**Figure 6:**
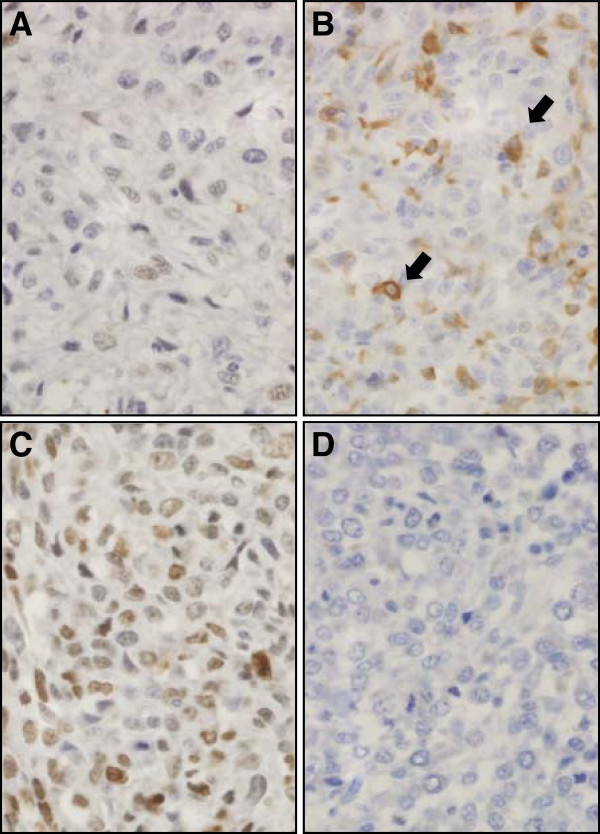
**Immunohistochemical analysis of nuclear HES1 protein expression in canine osteosarcoma.** Examples of low (**A** and **B**, score 2) and high (**C**, score 6) nuclear HES1 expression in canine osteosarcomas (**D** is a negative control treated only with secondary antibody). Panel **B** shows example of a field from a low scoring tumor (based on nuclear staining) that includes scattered strong cytoplasmic staining (arrows). All photomicrographs were taken at 40× magnification; haematoxylin counterstain.

Once we established that the RabMAb anti-human HES1 antibody provided specific targeting of HES1 protein in human cultured cells and FFPE tissues with good cross-reactivity in canine samples, we performed immunohistochemistry using canine primary OSA samples. Of the 20 tumor samples from the canine DFI > 300 and DFI < 100 tumor groups, 14 were scored as described in the methods (Figure 6). For six samples, IHC was not possible due to loss of tissue during processing or poor quality/quantity of staining/tissue present. All OSA samples evaluated with immunohistochemistry had variable positive staining for HES1 both across tumors and within tumors. The staining pattern of tumor cells was predominantly nuclear with diffuse cytoplasmic staining less common. The median HES1 reactivity score was 3 (range, 1 to 9). Of the 6 tumors from dogs with DFI > 300 days, 83.3% (n = 5) had a score of greater than 3, compared to only 25.0% (n = 2) of the 8 tumors from dogs with DFI < 100 days (Table 1). Consistent with our RT-qPCR results, average HES1 immunohistochemical staining was lower in tumors from dogs with DFI < 100 days, but because of low power did not reach statistical significance (Additional file 6).

**Figure 7:**
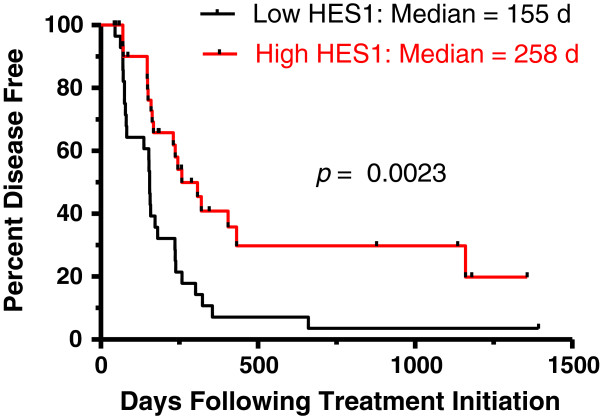
**High HES1 immunoreactivity score correlates with lower histologic grade and improved outcome in canine osteosarcoma.** Kaplan-Meier plot of disease free interval based on HES1 immunoreactivity score. Dogs with high HES1 scoring tumors (score ≥ 4, n = 28) had a statistically significantly longer disease free interval than dogs with low HES1 scoring tumors (score < 4, n = 33) (p = 0.0023, Log Rank test).

To further assess the utility of HES1 protein expression as a prognostic biomarker, we performed IHC on 61 primary canine OSA tissues from a subset of dogs in a previously reported prospective clinical trial [33]. Demographic information for this patient population is supplied in Additional file 7. IHC scores were assigned as described in materials and methods. HES1 was expressed in all tumors with a median HES1 immunoreactivity score of 4 in this population (range, 1 to 9). The overall median DFI was 168 (range 43 to 1,393+ days). The median DFI in dogs with a high HES1 immunoreactivity score (≥ 4) was 258 days compared to 155 days in dogs with a low HES1 immunoreactivity score (< 4) (p = 0.0023; Figure 7). Univariate analysis identified HES1, bone-specific alkaline phosphatase (BALP) activity, histologic grade, percent necrosis and mitotic index as potential predictors of DFI (Table 2, p < 0.1). Upon multivariate analysis, HES1, percent necrosis and mitotic index retained statistical significance (p = 0.029, 0.002 and 0.005 respectively; Table 2) as independent predictors of DFI. In summary, consistent with our prior RT-qPCR analysis, increased HES1 expression was identified as an independent prognostic biomarker for increased disease free survival in 61 canine OSAs treated by amputation and chemotherapy.

**Table 2:** Results of univariate/multivariate analysis of factors associated with clinical outcome

**Univariate analysis**					
		**Median DFI (d)**	**HR**	**P**	**95% CI**
**HES1 Score**	<4	155	0.388	0.0023	0.211-0.712
	≥4	258			
**BALP**	<36	273.5	1.871	0.0377	1.036-3.378
	≥36	157			
**Necrosis%**	<20%	239	1.799	0.098	0.897-3.609
	≥20%	168			
**Mitotic Index**	<54	258	3.234	0.0163	1.241-8.428
	≥54	153			
**Grade**	1 or 2	308	15.43	<0.0001	4.243-56.07
	3	75			
**Multivariate analysis**					
			**HR**	**P**	**95% CI**
**HES1 Score**			0.775	0.029	0.616-0.975
**Necrosis%**			1.032	0.002	1.012-1.053
**Mitotic Index**			1.033	0.005	1.01-1.057

*DFI* disease free interval, *BALP* bone-specific alkaline phosphatase.

## Discussion

Expression of *HES1* mRNA is frequently utilized as an indicator of Notch activity and Notch/HES1 activation has been implicated in a variety of human cancers with oncogenic activity in some tumor types and tumor suppressor activity in others [17-20,24-27]. The goals of this study were to evaluate expression of Notch receptors and signaling mediators, HES1 and HEY1, in canine OSA samples from dogs with DFI > 300 days and DFI < 100 days as well as samples of matched OSA and normal bone to explore associations with OSA progression and patient outcome. Gene array analysis focusing on 51 Notch/HES1 associated genes identified elevated expression of Notch signaling mediators in tumors relative to normal bone. We confirmed a statistically significant elevation of *NOTCH2*, *HEY1*, and *HES1* mRNA expression in OSA when compared with normal bone. Interestingly, we did not find elevated *HES1* expression in the most aggressive OSA when comparing good and poor responders, but instead identified a statistically significant association between high *HES1* mRNA and protein expression and longer DFI following standard treatment. Further, the gene array analysis of Notch/HES1 associated genes and RT-qPCR analysis of *NOTCH1, NOTCH2* and *HEY1* showed no significant differences in expression between the DFI groups. Overall, our findings indicate that alterations in Notch signaling occur during the development of canine OSA, but mechanisms that do not alter HES1 expression may drive the most aggressive tumors.

The oncogenic role of Notch signaling in OSA in humans is supported by previous studies [24-26]; however, the specific role of HES1 is less clear. A common finding regarding HES1 expression between these previous studies and ours is the variability of expression within human and canine OSA cells and tumors (please note for references 24 and 28, that data from experiments done using the OS187 or COL cell lines should be viewed with caution due to a recent disclosure that these cells are not OSA cells) [24-26,28]. For example, *HES1* mRNA expression in tumors relative to normal bone was elevated in 5 of 9 canine tumors relative to matched normal bone samples in our study (Figure 3B) and 6 of 10 human tumors in the Tanaka study [25]. There is also disagreement among studies as to which Notch receptors and target genes are functionally significant in OSA. Zhang et al. provided evidence that increased Notch1 activity and Notch1-induced expression of HES1 specifically are associated with invasion and metastasis in two OSA cell lines, the low HES1 expressing SAOS2 parental line and the metastatic, high HES1 expressing LM7 sub-line [24]. Inhibition of Notch signaling by a gamma-secretase inhibitor suppressed LM7 OSA cell invasion, but had no effect on proliferation or tumorigenesis; whereas induced expression of intracellular cleaved Notch1 (ICN1) or HES1 in the SAOS2 line increased invasiveness. Tanaka et al. identified elevations of *NOTCH2* and *HEY1* mRNA in human OSA biopsy specimens relative to normal bone, but *NOTCH1* and *HES1* mRNA expression was not consistently elevated. In the same study, treatment of OSA cells and tumors grown in nude mice with a gamma-secretase inhibitor reduced proliferation through a G1 block [25]. Differing results in these two studies may be due to different samples studied (tumor vs. cells) and/or the use of different gamma-secretase inhibitors. Our RT-qPCR data suggests that *NOTCH2* and *HEY1* may be primary mediators of Notch signaling in canine OSA as well. Interestingly, Zhang et al. observed both elevated *HES1* mRNA expression [24] and elevated HES1 protein expression [28] in the LM7 metastatic sub-line relative to the SAOS2 parent line. We also observed an increase in *HES1* mRNA expression in the MG63.2 metastatic sub-line relative to the MG63 parent line. However, western blot analysis identified similar levels of HES1 protein in the MG63 and MG63.2 lines suggesting that post-transcriptional regulation may be important.

Studies exploring the relationship between HES1 expression and patient outcome in OSA are limited. Our RT-qPCR results (n = 20) revealed significantly increased *HES1* mRNA expression in canine OSA from dogs with a longer DFI compared to those with a short DFI. This relationship was confirmed by immunohistochemical examination of HES1 protein in a larger dataset (n = 61). These results conflict with those of Hughes who conducted a RT-qPCR study using tissue from 16 primary OSAs that suggested lower *HES1* mRNA expression may be associated with a better prognosis [27]. Discrepancy from our results may be due to differing sample sizes, different measurements of outcome and different outcome groupings. Despite evidence of strong molecular similarities of canine and human OSA and high conservation of Notch/HES1 between species, there is also the possibility that canine tumors may exhibit different characteristics than their human counterparts. Until similar studies to evaluate nuclear immunoreactivity as a measure of protein expression are carried out in human tumors, no firm conclusions regarding possible differences in canine and human OSA with respect to HES1 expression can be made.

Previous studies examining HES1 expression in other cancers or during development provide candidate mechanisms for reduced HES1 expression in the presence of elevated Notch signaling: uncoupling of HES1 from Notch signaling, cell cycle regulation of HES1 expression, and post-transcriptional regulation. HES1 expression has been reported to be uncoupled from Notch signaling in Ewing’s sarcoma [15] and stimulation of HES1 transcription by sonic hedgehog (Shh) pathway occurs in mesodermal and neural stem cells [6 – 8]. Using RT-qPCR analysis, we identified significantly decreased SMO mRNA expression (p < 0.05) in the DFI < 100 tumors compared to the DFI > 300 tumors [32] suggesting that reduced HES1 expression in aggressive canine OSA might reflect a loss of Shh signaling. HES1 expression oscillations are both observed and necessary for cell cycle progression during neuronal development [45]; aggressive OSA tumor cells may utilize HES1 oscillatory patterns to manipulate the cell cycle and optimize their ability to metastasize and/or resist chemotherapy. Finally, several miRNAs have been shown to regulate HES1 (miR-124 and miR-23b) [46,47] and may contribute to altered HES1 expression in OSA cells and tumors.

In addition, HES1 protein may exhibit specific functions depending on its phosphorylation status and binding partners. Kannan et. al. found that interactions with HES1 stimulates PARP1 activation and cleavage, ultimately resulting in apoptosis in B-ALL (overall a tumor suppressor role for HES1) [20]. Further, in neuronal development, Ju et al. showed that HES1 interactions with phosphorylated PARP1 released HES1 from the HES1/groucho/TLE repressor complex and, upon HES1 phosphorylation, led to association with a co-activator complex, changing the role of HES1 from a transcriptional repressor to a transcriptional activator [48]. In bone development, via inhibition of RUNX2, Notch activity maintains a population of committed osteoblast precursors [49,50]. Interestingly, several studies also show that HES1 binding stabilizes and activates RUNX2 protein; thus, HES1 has been shown to both inhibit and enhance the activity of RUNX2 [49,51]. Additional studies exploring the phosphorylation status and binding partners of HES1 may provide a better understanding of these interactions in OSA.

## Conclusions

The results of the current study support the association of Notch pathway activation with the proliferative response of OSA. However, reduced HES1 expression in the most aggressive tumors despite the elevated expression of other Notch signaling effectors and targets indicates that HES1 is not an ideal sole surrogate marker of Notch signaling. Further, these findings suggest that additional mechanisms beyond Notch signaling may contribute to the aggressive phenotype of these tumors. Studies to define the role of Notch signaling in OSAs is warranted as inhibitors for this and other developmental pathways that impinge on HES1 are currently in clinical trials for the treatment of a variety of human cancers (summarized in Sang et al.) [52]. Research in this area may reveal important regulatory mechanisms contributing to metastasis and therapeutic resistance in both canine and human OSA. While we found that HES1 expression was not consistently linked to Notch signaling in canine OSA, our study has determined that reduced HES1 expression serves as an independent prognostic biomarker.

## Competing interests

The authors declare that they have no competing interests.

## Authors’ contributions

DDD carried out all mRNA and protein expression experiments (unless otherwise noted), scored IHC samples, analyzed data, performed statistical analyses (except for survival and regression analyses) and drafted the manuscript. KPA contributed to study design and carried out HES1 RT-qPCR for the DFI group tumors. LEP carried out sample preparation (RNA extraction from canine tissues and sectioning of FFPE canine tissues for IHC) and taught DDD and KPA RT-qPCR methodology including analysis of data. EJE provided guidance to DDD and JBC for IHC/ICC optimization and scoring. JBC assisted DDD with IHC and ICC optimization and scored IHC samples. TBB designed canine HES1 primers. DHT performed survival and regression statistical analyses. BEP graded histologic samples from the larger patient population. TJJ contributed to study design and provided canine HES1 primers. DLD conceived of the study design with TJJ, provided guidance and coordination for all experiments, and helped to draft the manuscript. All authors read and approved the final manuscript.

## Supplementary Material

Additional file 1 **Affymetrix Canine 2.0 microarray data processed with PLIER algorithm.** Selected Notch signaling pathway genes from Affymetrix Canine 2.0 microarray data including both previously published [35] and unpublished data (normal bone). Click here for file

Additional file 2 Sequences, amplicon sizes, and efficiencies of primer pairs used in RT-qPCR experiments. Click here for file

Additional file 3 **Western blot of MG63.2 and U20S whole cell, nuclear and cytosolic fractions for HES1.** A distinct band at 30 kDa is present in both MG63.2 and U2OS human OSA whole cell (W) and is enriched in nuclear extract (N) lysates. Larger non-specific bands predominate in the cytoplasmic fraction (C). Equal amounts of total protein were loaded in each lane. Click here for file

Additional file 4 **HES1 protein expression is not significantly different between MG63 and MG63.2 cell lines.** HES1 band intensity normalized to α-tubulin loading control. Bars represent mean +/- standard deviation from four independent experiments. Standard unpaired 2-tailed *t*-test was used to compare mean HES1 band intensity ratios for MG63 and MG63.2 Western blot. Click here for file

Additional file 5 **HES1 immunohistochemistry of control canine tissues.** Variably intense nuclear staining is present in bronchiolar epithelial cells (A) and in both exocrine and endocrine (islets cells, blue circle) pancreatic cells (C). B and D are the negative controls. All photomicrographs were taken at 40× magnification; haematoxylin counterstain. Click here for file

Additional file 6 **HES1 immunoreactivity in canine osteosarcomas from DFI < 100 and >300 groups.** Immunoreactivity scores of nuclear HES1 protein expression in tumor sections from DFI < 100 day (filled circles, n = 8) and DFI > 300 day (filled squares, n = 6) groups. Horizontal line and error bars are mean ± SEM (p = 0.1026). Click here for file

Additional file 7 **Summary demographic data for 61 canine patients from a previously reported clinical trial **[33]**.** Click here for file
