# Supplementary figures and images for: HES1, a target of Notch signaling, is elevated in canine osteosarcoma, but reduced in the most aggressive tumors
Source: BMC Vet Res. 2013 Jul 1;9:130. doi: 10.1186/1746-6148-9-130 (PMC3701487; doi:10.1186/1746-6148-9-130)

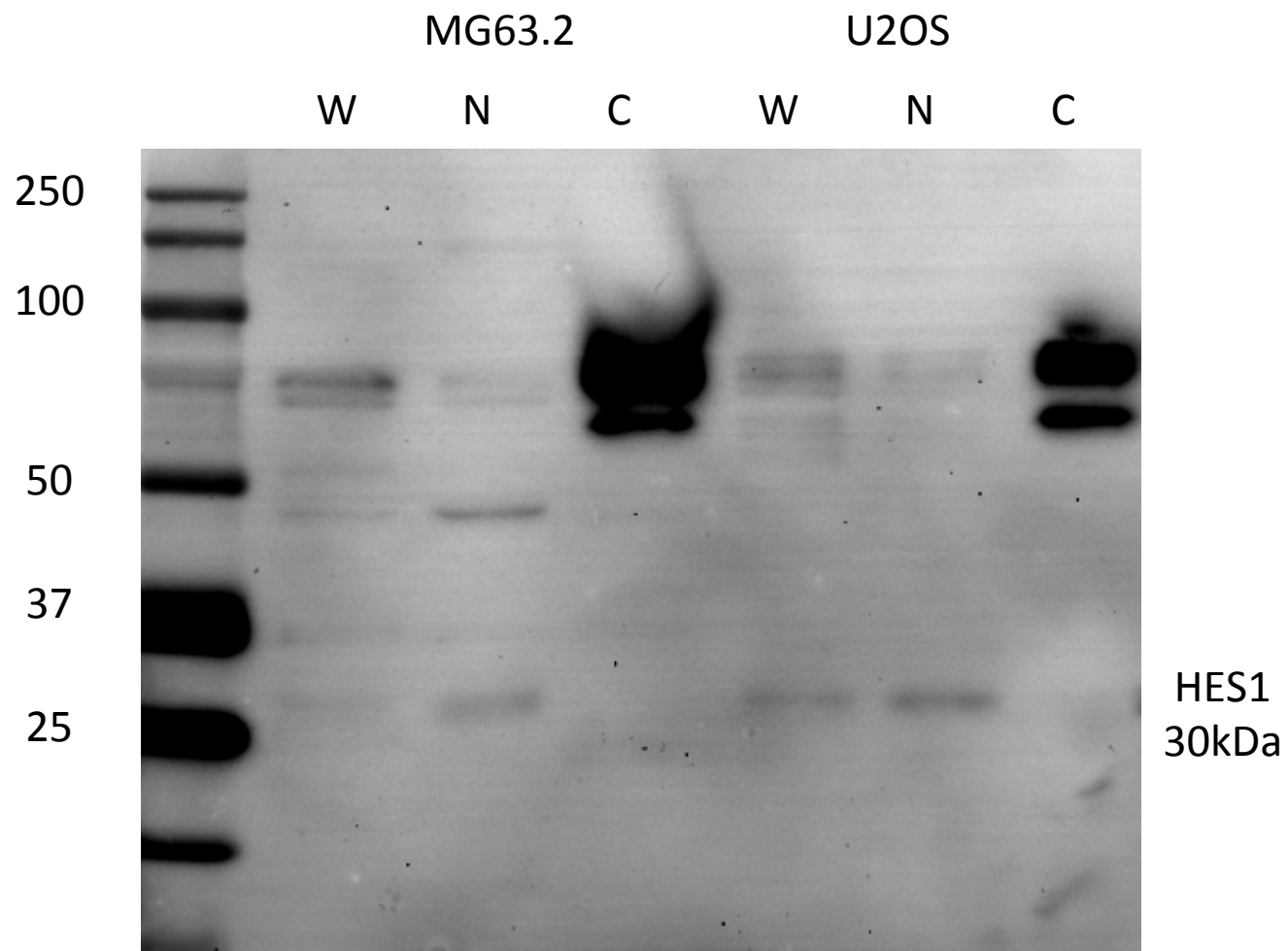

Supplement: Additional file 3 — Western blot of MG63.2 and U20S whole cell, nuclear and cytosolic fractions for HES1. A distinct band at 30 kDa is present in both MG63.2 and U2OS human OSA whole cell (W) and is enriched in nuclear extract (N) lysates. Larger non-specific bands predominate in the cytoplasmic fraction (C). Equal amounts of total protein were loaded in each lane. [file 1746-6148-9-130-S3.pdf]

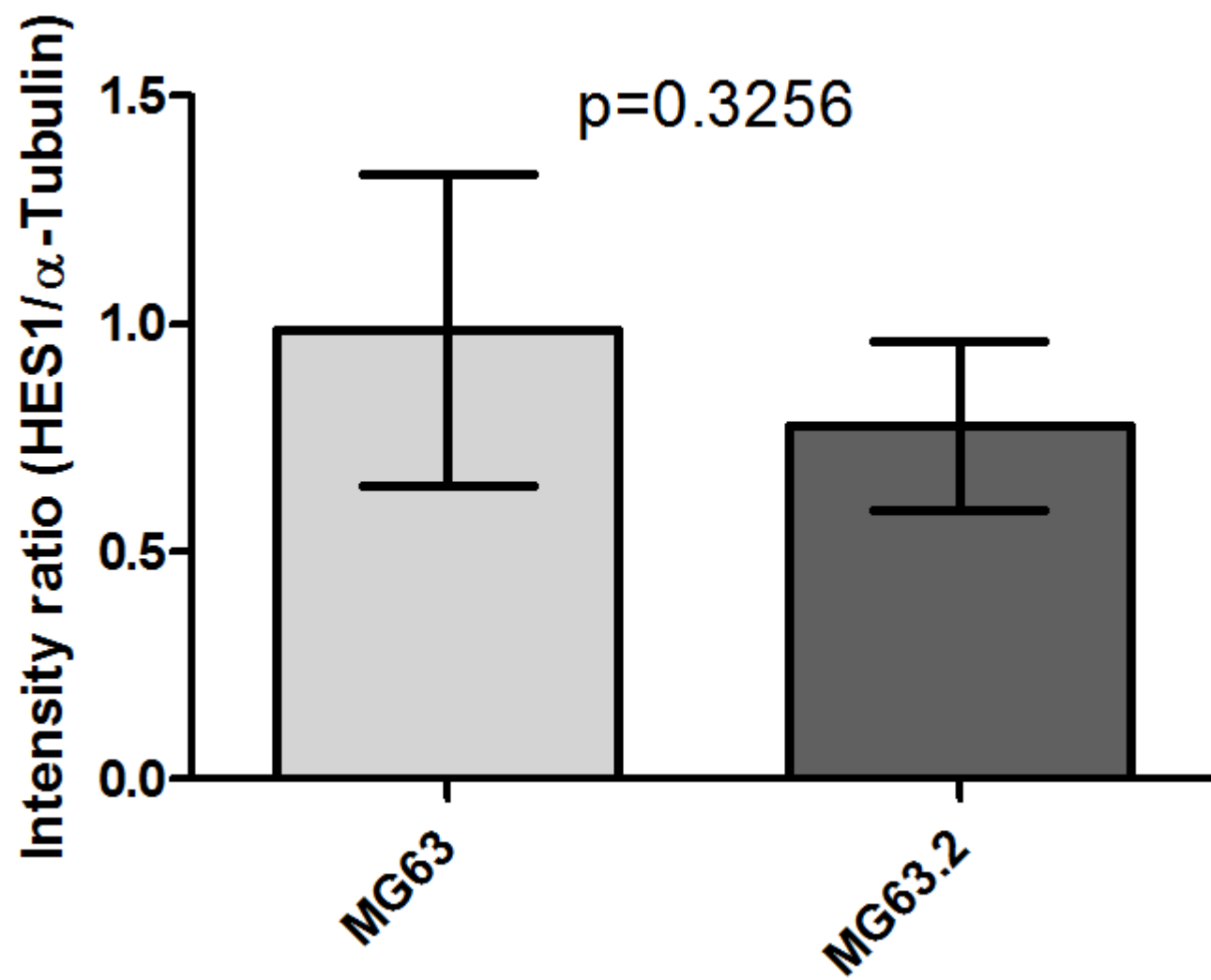

Supplement: Additional file 4 — HES1 protein expression is not significantly different between MG63 and MG63.2 cell lines. HES1 band intensity normalized to α-tubulin loading control. Bars represent mean +/- standard deviation from four independent experiments. Standard unpaired 2-tailed t-test was used to compare mean HES1 band intensity ratios for MG63 and MG63.2 Western blot. [file 1746-6148-9-130-S4.pdf]

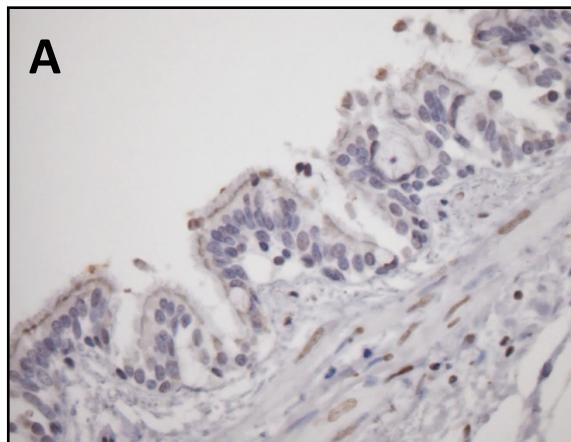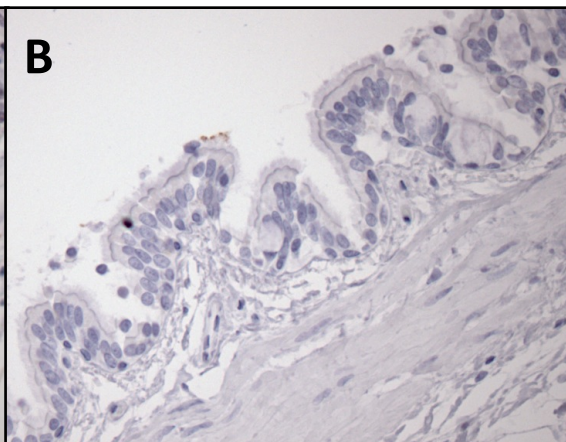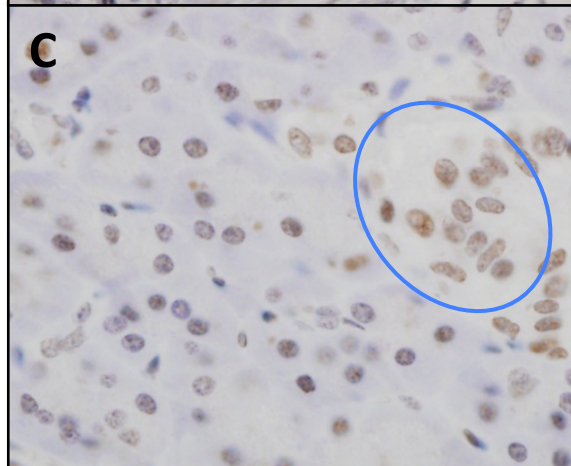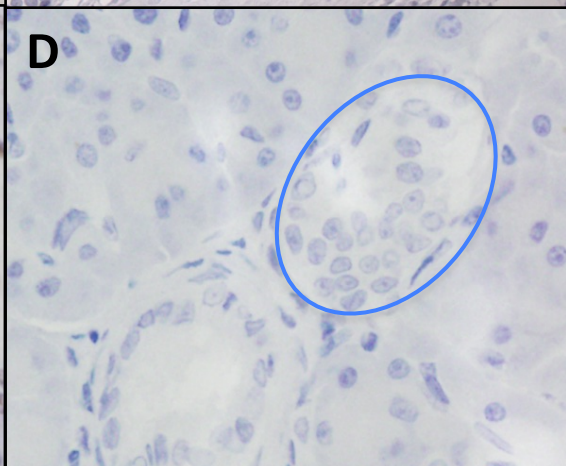

Supplement: Additional file 5 — HES1 immunohistochemistry of control canine tissues. Variably intense nuclear staining is present in bronchiolar epithelial cells (A) and in both exocrine and endocrine (islets cells, blue circle) pancreatic cells (C). B and D are the negative controls. All photomicrographs were taken at 40× magnification; haematoxylin counterstain. [file 1746-6148-9-130-S5.pdf]

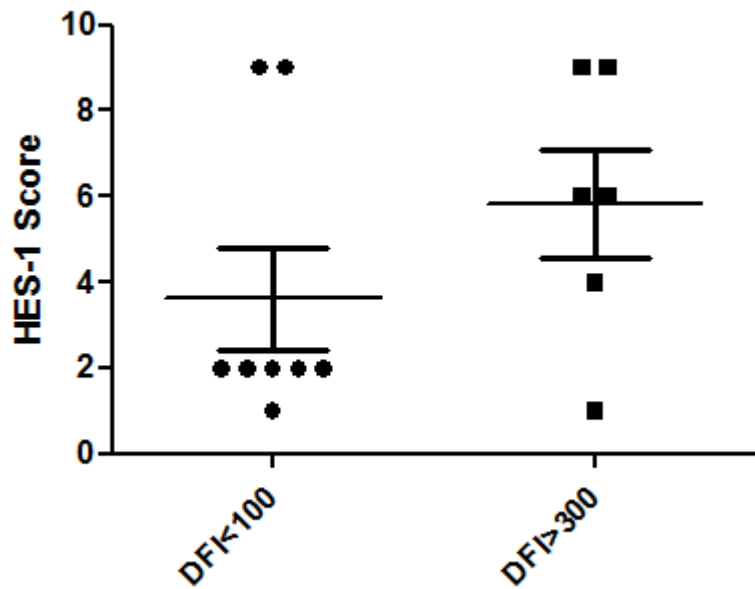

Supplement: Additional file 6 — HES1 immunoreactivity in canine osteosarcomas from DFI < 100 and >300 groups. Immunoreactivity scores of nuclear HES1 protein expression in tumor sections from DFI < 100 day (filled circles, n = 8) and DFI > 300 day (filled squares, n = 6) groups. Horizontal line and error bars are mean ± SEM (p = 0.1026). [file 1746-6148-9-130-S6.pdf]
